# An information transmission model for transcription factor binding at regulatory DNA sites

**DOI:** 10.1186/1742-4682-9-19

**Published:** 2012-06-06

**Authors:** Mingfeng Tan, Dong Yu, Yuan Jin, Lei Dou, Beiping Li, Yuelan Wang, Junjie Yue, Long Liang

**Affiliations:** 1 Beijing Institute of Biotechnology, Beijing, 100071, China; 2 Beijing Institute of Radiation Medicine, Beijing, 100850, China

## Abstract

**Background:**

Computational identification of transcription factor binding sites (TFBSs) is a rapid, cost-efficient way to locate unknown regulatory elements. With increased potential for high-throughput genome sequencing, the availability of accurate computational methods for TFBS prediction has never been as important as it currently is. To date, identifying TFBSs with high sensitivity and specificity is still an open challenge, necessitating the development of novel models for predicting transcription factor-binding regulatory DNA elements.

**Results:**

Based on the information theory, we propose a model for transcription factor binding of regulatory DNA sites. Our model incorporates position interdependencies in effective ways. The model computes the information transferred (*TI*) between the transcription factor and the TFBS during the binding process and uses *TI* as the criterion to determine whether the sequence motif is a possible TFBS. Based on this model, we developed a computational method to identify TFBSs. By theoretically proving and testing our model using both real and artificial data, we found that our model provides highly accurate predictive results.

**Conclusions:**

In this study, we present a novel model for transcription factor binding regulatory DNA sites. The model can provide an increased ability to detect TFBSs.

## Background

The transcription of genes is controlled by transcription factors (TFs), which bind to short DNA motifs that are known as transcription factor binding sites (TFBSs). Identification of TFBSs lies not only at the very heart of expanding our knowledge of regulatory elements in the genome by helping to decode genomic data, discover regulatory patterns in gene expression, and establish transcription regulatory networks, but also of explaining the origins of organismal complexity and development [1]. Computational identification of TFBSs is a rapid, cost-efficient way to locate unknown TFBSs. With increased potential for high-throughput genome sequencing, the availability of accurate computational methods for TFBS prediction has never been as important as it currently is. However, DNA regulatory elements are frequently short and variable, making the computational identification of them a challenging problem because the real TFBSs might be easily lost in random DNA sequences, i.e., the “background noise”.

To date, many models have been developed for transcription factor binding of regulatory DNA sites, and based on those models, numerous computational algorithms have been established to identify TFBSs. Several studies have utilised the structural information of DNA and protein to build predictive models for DNA binding sites [2-5]. These algorithms are able to identify previously uncharacterised binding sites for TFs and have improved performance over simple sequence profile models [6]. However, these algorithms have not been generally used because their parameters depend on the knowledge of the solved protein-DNA complex structures, which is a limited data set.

Several methods use pattern recognition algorithms derived from computer science or other research areas. These methods include support vector machines (SVMs) [7], self-organising maps (SOMs) [8], and Bayesian networks [9]. These algorithms can automatically provide objective and non-user-defined thresholds by training the programme with known data. Nevertheless, the biggest limitation of these methods might be the lack of explicitly biochemical or biophysical explanations.

Currently, position weight matrix (PWM) is the most common model for TFBS recognition. Many methods or programmes are based on the PWM model or its expansion, such as Match [10], the expectation–maximisation (EM) algorithm [11], and the stochastic variant of EM, the Gibbs sampling method [12,13]. In PWM, an *L*-long sequence motif is represented by a 4*L matrix, with weights giving the frequency of the four DNA bases (or the logarithm) in each of the *L* positions [6,14,15]. The basic PWM model is based on the biophysical considerations of protein–DNA interactions and uses the relative entropy, which is also known as the information content, as the criterion to determine whether an input sequence is a TFBS. According to this theory, the affinity between the factor and its TFBS is related to the free energy, which correlates with the relative entropy [6,14,15]. Therefore, in order for a sequence to be a TFBS, it must have higher relative entropy. Consequently, the relative entropy can be used as the criterion to detect a TFBS.

The PWM approach assumes that the contribution of each nucleotide position within a TFBS to the free energy is independent and that the effect on the binding strength is cumulative. We call this hypothesis the “independent hypothesis” because it supposes that each base of the motif is independent of the others. Methods based on the independent hypothesis are simple and have small numbers of parameters, making them easy to implement. These methods are widely used and often considered acceptable models for binding-site predictions.

The PWM model can suffer from high false-positive (FP) rates if motifs are degenerate. In addition, in some real cases, the affinity between factors and their TFBSs is weak, causing a high false-negative (FN) rate while using these methods. More importantly, the independent hypothesis can lead to deviations in the scoring mechanism and produce inaccurate results. Experimental evidence [16-20] suggests that there is interdependence among positions in the binding sites, which has prompted the development of models that incorporate position dependencies. The related methods include Bayesian networks [21], permuted Markov models [22], Markov chain optimisation [23], hidden Markov models [24], non-parametric models [25], and generalised weight matrix models [26]. Methods based on position-dependency models usually have better binding site prediction accuracy with lower FP rates. However, these methods require more complicated mathematical tools with more parameters to estimate and more experimental data than are typically available [27].

Orthogonal information from comparative genomics and information on co-regulation at the transcriptional level have also been integrated into these methods to identify cis-regulatory sites [28-31]. Methods have also been proposed to discover the composite regulatory module (CMA) [32,33]. Because most of these methods rely on the basic algorithms proposed previously, their performances are mainly determined by these basic algorithms.

Therefore, although significant progress has been made, the accuracy of the computational identification of TFBSs can still be improved. To tackle the general problem of binding site identification in the absence of high-throughput experimental data, theoretical models of binding sites are still required.

One aim of this work is to develop a new model that incorporates position interdependencies in effective ways to improve the computational prediction of TFBSs. Based on information theory [34,35], in this study, we propose a novel computational model. By theoretically proving and testing our model using both real and artificial data, we find that our model gives highly accurate predictive results.

### Information transmission model

#### An information transfer model for TFBS binding

In this paper, we treat the complex between a transcription factor and its binding site as a stand-alone system. During the binding process, energy exchanges occur between the TF and TFBS, and the spatial structure and physical state of the system change. We assume that the total amount of information in the system remains unchanged in this process and that information is only transferred between the factor and the site:

(1) INFO=IF1+IS1=IF2+IS2

**Figure 1:**
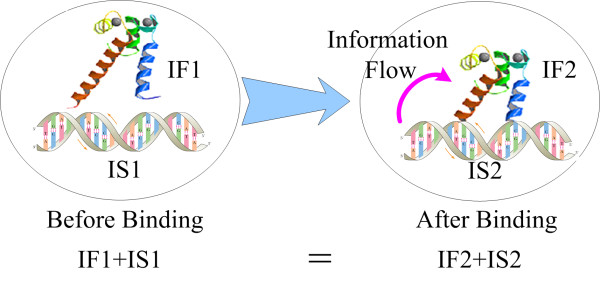
**Information transferred from TFBS to TF during binding.** In this figure, we assume the transcription factor and its binding site are a stand-alone system.

In this equation, *INFO* is the total information contained by the system, *IF*1 and *IF*2 are the information carried by the transcription factor in the unbound and bound states, respectively, and *IS*1 and *IS*2 are the information possessed by the DNA site at the unbound and bound states, respectively. During the binding process, the information flows from the TFBS to the factor (Figure 1). The transferred information is

(2) TI=IF2−IF1=IS1−IS2

Taking an *L*-bp sequence (*seq*) as the input sequence to be scanned, the *j*th base of *seq* is *seq*(*j*). The background probability of A, T, C, and G is *q*(*i*). In this formula, *i* represents the base A, T, C, or G, and the background probability can be obtained by scanning the chromosome sequences of the species. Before binding, the occurrence probability of base *seq*(*j*) is *q* (*seq*(*j*)). According to information theory [25,26], the information carried by the *jth* base is -log_2_(*q*(*seq*(*j*))). With the independent hypothesis, the total information carried by the input sequence can be simply calculated by summing all of the information carried by each base of *seq*:

(3) $$IS1=-\sum_{j=1}^{L}\log_{2}q\left(seq(j)\right)$$

Suppose that a transcription factor and its known TFBSs are aligned by an appropriate algorithm. In this study, we use *L* to represent the length of the aligned motif, *j* to represent the base position and *p*_*j*_(*i*) to represent the occurrence probability that the base *i* (A, T, C or G) appears at the position *j* according to the motif.

After the TF binding to its site, the state of the DNA sequence changes. The occurrence probability of base *seq*(*j*) changes to *p*_*j*_ (*seq*(*j*)); therefore, the information carried by the *jth* base becomes -log_2_*p*_*j*_ (*seq*(*j*)), and with the independent hypothesis, the total information IS2 is as follows:

(4) $$IS2=-\sum_{j=1}^{L}\log_{2}p_{j}\left(seq(j)\right)$$

The *TI* can be described as

(5) $$TI=IS1-IS2=\sum_{j=1}^{L}\log_{2}\frac{p_{j}\left(seq(j)\right)}{q\left(seq(j)\right)}$$

We hypothesise that a factor binds to a TFBS only if enough information is transferred from the site to the factor. We can use a basic criterion to determine whether the factor can bind to the sequence: the *TI* of the sequence must be larger than a threshold value. This value can be defined as the minimum transferred information (*MTI*), which is the natural and objective threshold used to determine whether the binding can occur. That is,

(6) $$threshhold\left(factor\right)=MTI\left(factor\right)=\min\left\{TI|TI=TI\left(TFBS\right),TFBS\in KnownTFBS\left(factor\right)\right\}$$

Once the *TI* of an input sequence is larger than *MTI*, then it is accepted as a possible TFBS.

### Enhancement of the model to be universal

The independent model might lead to inaccurate predictive results. In this section, we discuss in detail how this can happen by example and in theory and how we enhanced our model to be independent of this hypothesis.

**Figure 2:**
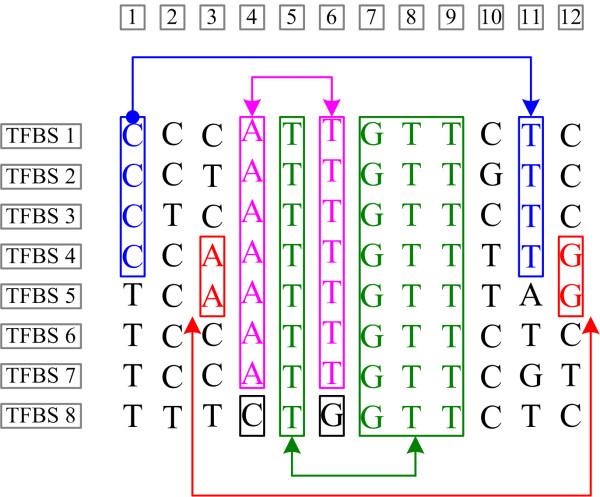
**Example of positive correlations.** The bases in the rectangles connected by the link are positively correlated. These eight known rox1 binding sites were taken from the promoter database of *Saccharomyces cerevisiae.*

An example of the correlation among different bases is shown in Figure 2. The same example is used by GuhaThakurta [1] to show the basic concept of the PWM and relative entropy methods. We can see that the 1^st^ and 11^th^ bases are correlated: when the 1^st^ base is C, the 11^th^ base is strictly T. We can find that P_1,11_(C,T) = 0.5, and P_1_ (C) P_11_(T) = 0.5*0.75 = 0.375. As P_1,11_(C,T)>P_1_ (C)P_11_(T); therefore, we conclude that these bases are positively correlated. When position 1 is C, there is a high probability that position 11 is T. For these two positions, P_1,11_(T,T) = 0.25, P_1_(T)P_11_(T) = 0.375. So P_1,11_(T,T)<P_1_ (T)P_11_(T); therefore, we conclude that the positions are negatively correlated, which means that when position 1 is T, there is a high probability that position 11 is not T. Such correlations are not rare, as they can be found in most of the real TFBS data set.

Based on this observation, we propose a formal definition of positive and negative correlations of the bases in a motif: if $P_{seq\left(i_{1}\right),\cdots ,seq\left(i_{m}\right)}>P_{seq\left({i^{\prime }}_{1}\right),\cdots ,seq\left({i^{\prime }}_{k}\right)}*P_{seq\left({i^{\prime }}_{k+1}\right),\cdots ,seq\left({i^{\prime }}_{m}\right)}$, then $seq\left({i^{\prime }}_{1}\right),\cdots ,seq\left({i^{\prime }}_{k}\right)$ and $seq\left({i^{\prime }}_{k+1}\right),\cdots ,seq\left({i^{\prime }}_{r}\right)$ are positively correlated. If they are equal, then $seq\left({i^{\prime }}_{1}\right),\cdots ,seq\left({i^{\prime }}_{k}\right)$ and $seq\left({i^{\prime }}_{k+1}\right),\cdots ,seq\left({i^{\prime }}_{r}\right)$are independent; otherwise, they are negatively correlated. In this formula, seq(*i*) is the *i*th base of the sequence *seq.* For example, the 4^th^ and 6^th^ bases are positively correlated and contain no more or less information than only the individual base. Therefore, the independent hypothesis leads to an inaccurate estimation of the *TI*, thereby making an erroneous prediction of the TFBS. Similarly, the use of other methods that are based on the independent hypothesis also results in incorrect scores and leads to inaccurate predictive results. To avoid this inaccuracy, the model was enhanced to address the correlations such that it is capable of determining the correct *TI* despite the inaccuracy of the independent hypothesis.

First, we know that after binding of the TF to the TFBS, the information encoded by the TFBS *seq*, is$IS2=I_{L}=-\log_{2}p_{\mathrm{seq}}=-\log_{2}p_{seq\left(1\right)}{}_{,\cdots ,seq\left(L\right)}$. In this equation, $p_{\mathrm{seq}}=p_{seq\left(1\right)}{}_{,\cdots ,seq\left(L\right)}$ is the occurrence probability of $seq=seq\left(1\right),\cdots ,seq\left(L\right)$ versus all of the TFBSs of the TF. Due to unknown TFBSs and lack of statistical data of the known TFBSs, we cannot determine *p*_*seq*_ or *I*_*L*_ directly, but these terms can be estimated from the known TFBSs.

We use the information of *r-*base sub-sequences $seq\left(i_{1}\right),\cdots ,seq\left(i_{r}\right)\left(i_{1}>i_{2}>\cdots >i_{r}\right)$ to estimate the information of the full sequence. The probability of a *r-*base sub-sequence,$p_{seq\left(i_{1}\right),\cdots ,seq\left(i_{r}\right)}$, can be approximated as ${\tilde{p}}_{seq\left(i_{1}\right),\cdots ,seq\left(i_{r}\right)}$ by investigating the known TFBS, and in the following steps, we assume that $p_{seq\left(i_{1}\right),\cdots ,seq\left(i_{r}\right)}$ and ${\tilde{p}}_{seq\left(i_{1}\right),\cdots ,seq\left(i_{r}\right)}$ are the same. Therefore, $-\log_{2}P_{seq\left(i_{1}\right),\cdots ,seq\left(i_{r}\right)}$ is the information of the sub-sequence.

This probability can reveal the correlation among these bases. For example, if base *i*_1_ is fully and positively correlated with base *i*_*k*_, as the 4^th^ and 6^th^ positions in Figure 2 are, then $p_{seq\left(i_{1}\right)seq\left(i_{k}\right)}=p_{seq\left(i_{1}\right)}=p_{seq\left(i_{k}\right)}>p_{seq\left(i_{1}\right)}p_{seq\left(i_{k}\right)}$; hence, the information of these two bases is $I_{i_{1},i_{k}}=-\log_{2}P_{seq\left(i_{1}\right)}=\frac{1}{2}I_{\mathrm{independent}}$ (i.e., it is only half of the information of the independent situation). If base *i*_1_ is independent from base *i*_*k*_, then $p_{seq\left(i_{1}\right)seq\left(i_{k}\right)}=p_{seq\left(i_{1}\right)}\times p_{seq\left(i_{k}\right)}$ and $I_{i_{1},i_{k}}=-\log_{2}\left(P_{seq\left(i_{1}\right)}\times P_{seq\left(i_{k}\right)}\right)=I_{\mathrm{independent}}$.

As there are $C_{L}^{r}$ such *r*-base sub-sequences in total, the average information for *r*-base sub-sequences is $\frac{-1}{C_{L}^{r}}\sum_{i_{1}>i_{2}>\cdots >i_{r}}\log_{2}P_{seq\left(i_{1}\right),\cdots ,seq\left(i_{r}\right)}$. Because the length of these sub-sequences is *r*, then the average information carried by one base is $\frac{-1}{rC_{L}^{r}}\sum_{i_{1}>i_{2}>\cdots >i_{r}}\log_{2}P_{seq\left(i_{1}\right),\cdots ,seq\left(i_{r}\right)}$. The information of the whole sequence can be estimated by simply multiplying by the length *L*:

(7) $$I=I_{L}\approx I_{r}\approx {\tilde{I}}_{r}=\frac{-L}{rC_{L}^{r}}\sum_{i_{1}>i_{2}>\cdots >i_{r}}\log_{2}{\tilde{p}}_{seq\left(i_{1}\right),\cdots ,seq\left(i_{r}\right)}$$

Similar to the example in Figure 2, if there is a strong tendency for the bases to be positively correlated, then it can be assumed that

(8) $$p_{seq\left(i_{1}\right)}{}_{,\cdots ,seq\left(i_{r}\right)}>p_{seq\left({i^{\prime }}_{1}\right)}{}_{,\cdots ,seq\left({i^{\prime }}_{k}\right)}p_{seq\left({i^{\prime }}_{k+1}\right)}{}_{,\cdots ,seq\left({i^{\prime }}_{\text{r}}\right)}$$

Under this assumption, we can prove an important relationship, as follows:

(9) $$I_{r+1}<I_{r}$$

From (8) we know that

(10) $$\begin{array}{l} I_{r}=\frac{-L}{rC_{L}^{r}}\sum_{i_{1}>i_{2}>\cdots >i_{r}}\log_{2}p_{seq\left(i_{1}\right)}{}_{,\cdots ,seq\left(i_{r}\right)}\\ =\frac{-L}{rC_{L}^{r}}\sum_{i_{1}>i_{2}>\cdots >i_{r}}\frac{1}{C_{r}^{x}}\left(C_{r}^{x}\right)\log_{2}p_{seq\left(i_{1}\right)}{}_{,\cdots ,seq\left(i_{r}\right)}\\ =\frac{-L}{rC_{L}^{r}}\sum_{i_{1}>i_{2}>\cdots >i_{r}}\frac{1}{C_{r}^{x}}\sum_{i}^{C_{r}^{x}}\log_{2}p_{seq\left(i_{1}\right)}{}_{,\cdots ,seq\left(i_{r}\right)}\\ <\frac{-L}{rC_{L}^{r}}\sum_{i_{1}>i_{2}>\cdots >i_{r}}\frac{1}{C_{r}^{x}}\sum_{i^{\prime }}^{C_{r}^{x}}\left(\log_{2}p_{seq\left({i^{\prime }}_{1}\right)}{}_{,\cdots ,seq\left({i^{\prime }}_{x}\right)}+\log_{2}p_{seq\left({i^{\prime }}_{x+1}\right)}{}_{,\cdots ,seq\left({i^{\prime }}_{r}\right)}\right)\\ =\frac{-L}{rC_{L}^{r}}\left(\frac{1}{C_{r}^{x}}\frac{C_{r}^{x}C_{L}^{r}}{C_{L}^{x}}\sum_{i_{1}>i_{2}>\cdots >i_{x}}\log_{2}p_{seq\left(i_{1}\right)}{}_{,\cdots ,seq\left(i_{x}\right)}+\frac{1}{C_{r}^{x}}\frac{C_{r}^{r-x}C_{L}^{r}}{C_{L}^{r-x}}\sum_{i_{1}>i_{2}>\cdots >i_{r-x}}\log_{2}p_{seq\left(i_{1}\right)}{}_{,\cdots ,seq\left(i_{r-x}\right)}\right)\\ =\frac{-L}{r}\left(\frac{1}{C_{L}^{x}}\sum_{i_{1}>i_{2}>\cdots >i_{x}}\log_{2}p_{seq\left(i_{1}\right)}{}_{,\cdots ,seq\left(i_{r}\right)}+\frac{1}{C_{L}^{r-x}}\sum_{i_{1}>i_{2}>\cdots >i_{r-x}}\log_{2}p_{seq\left(i_{1}\right)}{}_{,\cdots ,seq\left(i_{r-x}\right)}\right)\\ =\frac{-L}{r}\left(\frac{x}{L}\frac{L}{xC_{L}^{x}}\sum_{i_{1}>i_{2}>\cdots >i_{x}}\log_{2}p_{seq\left(i_{1}\right)}{}_{,\cdots ,seq\left(i_{r}\right)}+\frac{r-x}{L}\frac{L}{\left(r-x\right)C_{L}^{r-x}}\sum_{i_{1}>i_{2}>\cdots >i_{r-x}}\log_{2}p_{seq\left(i_{1}\right)}{}_{,\cdots ,seq\left(i_{r-x}\right)}\right)\\ =\frac{x}{r}I_{x}+\frac{r-x}{r}I_{r-x} \end{array}$$

Next, we obtain

(11) $$I_{r}<\frac{x}{r}I_{x}+\frac{r-x}{r}I_{r-x}$$

According to (11), we can infer that

(12) $$I_{r+1}<\frac{r}{r+1}I_{r}+\frac{1}{r+1}I_{1}<\frac{r-1}{r+1}I_{r-1}+\frac{2}{r+1}I_{1}<\cdots <I_{1}$$

If we assume that $I_{r+1}\geq I_{r}$, then we immediately obtain $I_{r+1}\geq I_{1}$, which contradicts (11). Therefore, it must be the case that $I_{r+1}<I_{r}$. Hence, (10) is proved.

Immediately, we know that when the correlation is positive

(13) $$I_{L}<I_{r}<I_{\mathrm{independent}}\left(2\leq r\leq L-1\right)$$

Similarly, if the correlation tends to be negative, then the following must be true:

(14) $$I_{r+1}>I_{r}$$

Therefore,

(15) $$I_{L}>I_{r}>I_{\mathrm{independent}}\left(2\leq r\leq L-1\right)$$

If the TFBS *seq* conforms to the independent hypothesis, according to (5) and (8), its information is

(16) $$I_{\mathrm{independent}}\equiv -\sum_{i=1}^{L}\log_{2}P_{seq\left(i\right)}\equiv I_{1}$$

Therefore, conversely, the tendency of *I*_*r*_ can be used to judge if the correlation is positive, negative, or independent. We now know that *I*_*independent*_ would overestimate (when the correlation is positive) or underestimate (when the correlation is negative) the *I*_*L*_ if the independent hypothesis is not true. Again, this finding can explain why using the independent hypothesis can lead to inaccurately predicted results. More importantly, from (13) and (15), we know that *I*_*r*_ is more accurate than *I*_*independent*_ when *r* ≥ 2. So, we can use *I*_*r*_ (*r* ≥ 2) to estimate the information and obtain the predictive results with more accuracy.

The method for calculating the background probabilities must be revised accordingly to adapt to the enhanced model. Instead of counting each single base by scanning the chromosome sequences to obtain the background probability under the independent hypothesis, a window of length *L* slides through the chromosomes, and all of the *r*-base sub-sequences in this window are counted. After the scanning, 4^r^ probabilities are calculated for all of the 4^r^ possible *r*-base sub-sequences. These values are used to estimate the information carried by the TFBS before the binding event:

(17) $$IS1\left(seq\right)=IS1{\left(seq\right)}_{L}\approx IS1{\left(seq\right)}_{r}=\frac{-L}{C_{L}^{r}r}\sum_{i_{1}>i_{2}>\cdots >i_{r}}\log_{2}q_{seq\left(i_{1}\right),\cdots ,seq\left(i_{r}\right)}$$

In this equation, $q_{seq\left(i_{1}\right),\cdots ,seq\left(i_{r}\right)}$ is the background correlation probability, calculated as described previously.

In addition, the formula for estimating the transferred information is changed as follows:

(18) $$TI_{r}=IS1{\left(seq\right)}_{r}-IS2{\left(seq\right)}_{r}=\frac{L}{rC_{L}^{r}}\sum_{i_{1}>i_{2}>\cdots >i_{r}}\log_{2}\frac{p_{seq\left(i_{1}\right),\cdots ,seq\left(i_{r}\right)}}{q_{seq\left(i_{1}\right),\cdots ,seq\left(i_{r}\right)}}$$

Once *TI*_*r*_ ≥ *MTI*_*r*_ (*factor*), then *seq* is accepted as a possible TFBS.

## Results

### Performance in *Saccharomyces cerevisiae* promoter regions

We tested our model by calculating the *TI* for all of the known TFBSs of 10 well-characterised transcription factors in the yeast *S. cerevisiae* promoter database (SCPD) [36]. We found that most of the TFBSs have a *TI* larger than 0. This evidence strongly supports our TI hypothesis that the information is transferred from the TFBS to the factor, and binding of the TF to the TFBS only happens if enough information is transferred.

**Figure 3:**
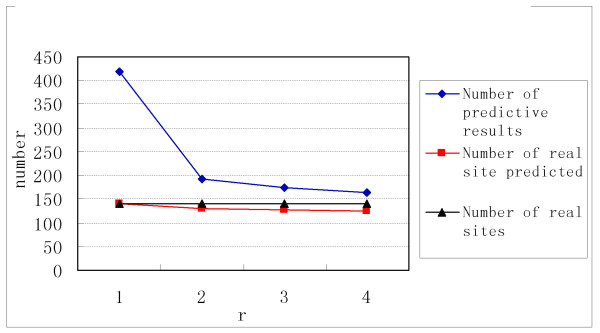
**The number of predicted TFBS.** With 100% of the known TFBSs as the training set, the number of predicted TFBS decreases more than two times when r changes from 1 to 2, which guarantees an increase in accuracy.

**Figure 4:**
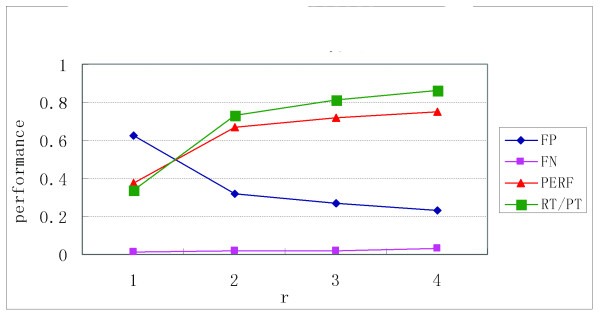
**Variety of performance as r changes from 1 to 4. In this figure, the proportion of the training set = 100%; however, the figures for proportion of training set = 25%, 75% and 100% are similar.** In this figure, PERF=(k P)/(K P), where K is the set of known motif sites and P is the set of predicted motif sites. RT/ PT is defined as the ratio of the real TFBSs to the predicted TFBSs.

First, we use 100% of the known TFBSs as the training set to work out the *MTI* for each TF and test our method with r = 1, 2, 3, and 4 on this data set. We observed that the number of predicted TFBS decreases more than twofold when *r* changes from 1 to 2 (Figure 3), which guarantees an increase in accuracy as Figure 4 shows. From Figure 3 and Figure 4, we observe that when *r* ≥ 2, the performance increases with increasing *r*-values but not as significantly as when *r* changes from 1 to 2. Because the computational complexity of our method rapidly increases as *r* increases, *r* = 3 is a proper value to obtain good performance and maintain a low level of computational complexity. Therefore, the results of *r* = 3 were used to compare this method with the others.

**Figure 5:**
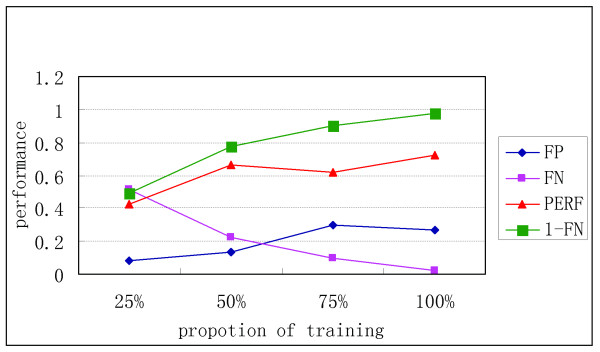
**Variety of average performance as the proportion of the training set changes from 25% to 100%, where x=y. In this figure, r = 3; however, the figures for r = 1, 2, and 4 are similar.** The definition of PERF is same as in SI Figure 4. The figure of our TI model is powerful when little is known about the TFBS. For example, 49% of TFBSs are identified when trained by 25% known TFBSs.

Next, we examined how the average performance changes as the proportion of the training set increased from 25% to 100% with r = 3 (Figure 5). We found that as the proportion increased, 1-FN increased linearly; hence, more of the real TFBSs were identified. Moreover, this curve indicated that the method is powerful when little is known about the TFBS. For example, 49% of TFBSs were identified when the model was trained by 25% known TFBSs. Additionally, the FP rate increased little when the proportion of the training set increased, and it was always below 0.3.

In this study, we illustrate several snapshots of TI by scanning several sequences of *S. cerevisiae*. These sequences cover the coding regions, the regulatory regions and the “flank” regions.

**Figure 6:**
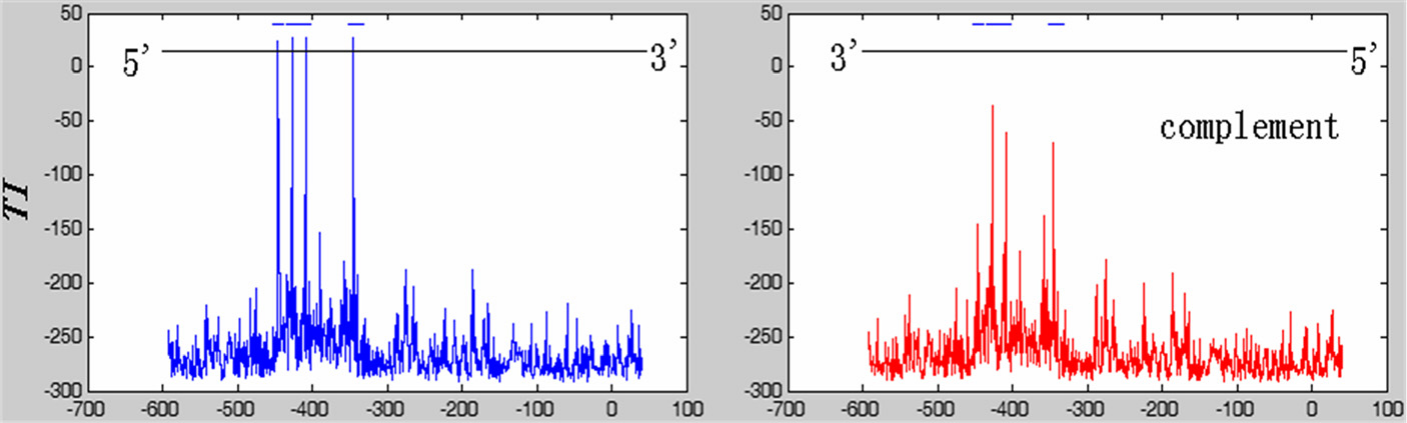
**Snapshots 1 of TI.** Factor=al4, ORF=YBR020W, r = 3, proportion of training set = 75%. The short lines on the top mark the position of the real TFBS, and the red lines are TI values from the complementary strand; the long lines in the middle denote the MTI of the specific factor. There are 4 TFBS on the strand from 5’ to 3’, and the highest peaks are almost precisely the real TFBSs.

In Figure 6, we illustrate a snapshot of *TI* by scanning a promoter region of *S. cerevisiae*. With 75% of the real TFBSs as the training set, we obtained the *MTI* for the factor. The highest peaks are precisely the real TFBSs, and there are also peaks on the opposite strand that do not reach the threshold.

### A comparison of the TI model with other methods

To illustrate the performance of the information transmission model, we implemented this novel model with a programme named tfbsInfoScanner and compared it with commonly used motif identification programmes, such as SOMBRERO, MEME and AlignACE. Mahony et al. [17] proposed the TFBS prediction method SOMBRERO and compared the results derived from SOMBRERO with those from two popular motif finding programmes, MEME [37] and AlignACE [11]. These researchers used the same real data set that we used. To efficiently analyse the performance of our method and to avoid repetitive and time-consuming computation, we used the same real sequence data set and compared results derived from our method to those obtained from SOMBRERO, MEME and AlignACE.

Table 1 shows a performance comparison of our method and three other programmes. The results indicate that when the proportion of the training set is larger than or equal to 50%, our method achieves the best performance in most cases.

**Table 1:** **Performance comparison between our TI method ( *r* ** **= 3) and three other programmes: SOMBRERO, MEME and AlignACE**

**Factor**		** *abf1* **	** *csre* **	** *gal4* **	** *gcn4* **	** *gcr1* **	** *hstf* **	** *mat* **	** *mcb* **	** *mig1* **	** *pho2* **
SOMBRERO	FP	0.56	0.727	0.235	0.286	0.69	0.571	0.25	0.645	0.68	0.909
	FN	0.45	0.25	0.071	0.6	0.222	0.111	0.308	0.083	0.2	0.5
MEME	FP	0.182	0.667	0.167	0.8	0.444	0.75	0.267	0.25	1	1
	FN	0.55	0.5	0.286	0.92	0.444	0.333	0.154	0.25	1	1
AlignACE	FP	0.375	0.824	0.083	0.444	0.625	0.556	0	0.083	0.909	1
	FN	0.5	0.25	0.214	0.6	0.333	0.111	0.308	0.083	0.9	1
TI model with	FP	** 0 **	** 0 **	** 0 **	** 0.182 **	** 0.333 **	** 0 **	** 0 **	** 0 **	** 0 **	** 0 **
25% known TFBS as training set	FN	0.727	0.5	0.643	** 0.259 **	0.692	0.667	0.526	0.333	0.429	** 0.5 **
TI model with	FP	** 0 **	** 0.333 **	** 0 **	** 0.226 **	** 0.143 **	** 0 **	0.294	** 0 **	** 0 **	** 0 **
50% known TFBS as training set	FN	**0.455**	** 0 **	0.286	** 0.037 **	0.308	0.5	**0.158**	** 0.083 **	**0.214**	** 0.375 **
TI model with	FP	** 0 **	** 0 **	** 0 **	** 0.25 **	0.615	0.783	0.25	** 0 **	** 0 **	** 0.25 **
75% known TFBS as training set	FN	** 0.182 **	** 0 **	0.143	** 0.037 **	** 0.077 **	** 0 **	**0.158**	** 0 **	** 0.143 **	** 0.125 **
TI model with	FP	** 0 **	** 0 **	** 0 **	** 0.265 **	0.577	** 0.526 **	*0.222*	** 0 **	** 0 **	** 0.571 **
100% known TFBS as training set	FN	** 0 **	** 0 **	** 0 **	** 0 **	** 0 **	0.167	** 0.053 **	** 0 **	** 0.071 **	** 0 **

The best performances of the other three programmes are underlined. The performances of our method that are better than the best of the other three programmes are in bold and underlined. The performances that were close to the best of the other three programmes are in bold. The results show that when the proportion of the training set is larger than or equal to 50%, our method achieves the best performance in most cases.

### Performance on artificial sequences

To examine the performance of our method in discovering “unknown” TFBSs, we subsequently trained our method with all of the known TFBSs and embedded the artificial sequences with pseudo-motifs. Similar to Mahony et al. [17], we also generated three artificial test set, although using our own method. In the artificial test set used by Mahony et al., each set comprises 10 data sets, each of which comprises 10 sequences; each sequence harbours a random number of occurrences (0 ~ 3) for each of the binding motifs for *gcn4gal4* and *mat1* (generated from PWMs). The total lengths of these three sets of 100 sequences are 4500, 8000 and 12500 bp, respectively. The average length of one sequence is therefore 45 bp, 80 bp or 125 bp, but each sequence harbours at most 9 occurrences of the motifs. We believe this number of occurrences may be too dense, and perhaps a high occurrence of pseudo-TFBSs may be encoded by these sequences.

In our modified method, we also generated three artificial test sets with different sequence lengths (450, 800 and 1250 bp), and each test set consists of 10 sequences that were randomly generated according to the GC content of *S. cerevisiae*. Each sequence harbours a random number of occurrences (0 ~ 3) for each of the binding motifs for *gcn4gal4* and *mcb* (randomly generated from PWMs). Mahony et al. [17] used *mat1* as a test object, but in the new version of SCPD, the TFBS of *mat1* is split into *mat1_alpha* and *mat1_beta*; therefore, we arbitrarily chose *mcb* as a substitute for *mat1*. This test set is more rigorous because these artificial sequences are 10 times longer, leading to an increase in the number of random sequences, which may result in a higher FP rate. As our method is still under development, in this test, the pseudo-TFBSs are also generated from the PWMs. Because the PWM method assumes that the independent hypothesis is true, these pseudo-TFBSs cannot correctly indicate correlation among the bases. This deficiency might lead to a lower *TI*, and, therefore, some pseudo-TFBSs may not be identified by our method. However, we can investigate what happens when scanning these artificial sequences.

**Figure 7:**
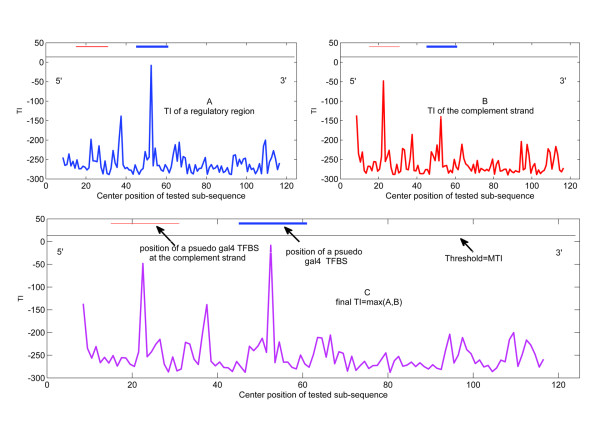
**A typical TI spectrum of the artificial regulatory region harbours unidentified pseudo-TFBSs.** The *TI* spectrum is on the bottom, and the *TI* of the complementary side is on the right. In this figure, the horizontal axis indicates the centre of the input sub-sequence with length that equals the aligned TFBSs of the specific factor. The data shows two embedded pseudo-gal4 TFBSs in the sequence. We can see that there are two *TI* peaks that are distinct but do not reach the threshold.

The average performance in each test set using *r* = 3 is summarised in Table 2. As demonstrated, most pseudo-TFBSs of *gcn4* and *mcb* were recognised. As for *gal4*, almost none of these sites was identified. Almost none of the unreal result was predicted at the same time. This result was observed mainly because the correlations of actual TFBSs are strong, while the pseudo-motifs do not have such correlations, and therefore their *TI* is far below the *MTI*. However, this finding does not mean that our method is ineffective in identifying pseudo-TFBSs. A typical snapshot of the artificial regulatory region that harbours the unrecognised pseudo-TFBSs is shown in Figure 7. Although these pseudo-TFBSs have no correlation in their sites, our method can still identify a strong *TI*.

**Table 2:** **Average performance of the artificial sequence data set (r = 3), perf = (kz∩P)/(K∪P), where K is the set of known motif sites and P is the set of predicted motif sites**[30]

**Length**	**Index**	** *gcn4* **	** *gal4* **	** *mcb* **	**Average**
	**FP**	0.75	0	0.647	0.466
450*10	**FN**	0.083	1	0.143	0.409
	**perf**	0.244	0	0.333	0.192
	**PT/ RT**	3.667	0	2.429	2.032
	**FP**	0.892	0	0.75	0.547
800*10	**FN**	0.2	1	0.333	0.511
	**perf**	0.105	0	0.222	0.109
	**PT/ RT**	7.4	0	2.667	3.356
	**FP**	0.936	0	0.756	0.564
1250*10	**FN**	0.25	0.875	0.286	0.470
	**perf**	0.063	0.143	0.222	0.143
	**PT/ RT**	11.75	0.143	2.929	4.941

In this table, PT/ RT is defined as the ratio of predicted TFBS versus the pseudo-TFBSs.

The average performance for *r* = 1 is also summarised in Table 3. When *r* = 1, our method is equivalent to the assumption that the independent hypothesis is true; therefore, all of the pseudo-TFBSs were identified. Not surprisingly, the FP rate was high. According to both sets of results, the FN rate was low. The FP rate was maintained at a moderate level for *r* = 3, even though these artificial sequences were 10 times longer than those described previously.

**Table 3:** Average performance of the artificial sequence data set (r = 1)

**Length**	**Index**	** *gcn4* **	** *gal4* **	** *mcb* **	**average**
	**FP**	0.894	0.308	0.917	0.706
450*10	**FN**	0	0	0	0
	**perf**	0.098	0.692	0.073	0.288
	**PT/ RT**	10.25	1.444	13.714	8.469
	**FP**	0.953	0.176	0.943	0.691
800*10	**FN**	0	0	0	0
	**perf**	0.047	0.824	0.057	0.309
	**PT/ RT**	21.1	1.214	17.583	13.299
	**FP**	0.975	0.125	0.951	0.684
1250*10	**FN**	0.125	0	0	0.042
	**perf**	0.024	0.875	0.049	0.316
	**PT/ RT**	35.625	1.143	20.286	19.018

## Discussion

During evolution, regulatory instructions or information were encoded in the DNA sequence. Redundant coding (or correlated coding) is utilised to ensure that the important regulatory information will be inherited and transferred correctly. During the binding process, the transcription factor reads the regulatory instructions from the TFBS and subsequently guides transcription according to the regulatory information. In other words, the factor reading the special regulatory instruction from the TFBS then instructs the transcription according to the regulatory information obtained from the TFBS. Nucleic acids needed to be coded in a redundant manner to ensure that the regulatory information can be transferred correctly, and therefore these sites are not independent of the others.

With our model, for the sequences encoding motifs, such as TFBSs, the input sequences can be scanned, and the sub-sequences for which the *TI* is greater than the *MTI* of the motif can be taken as the predictive hits.

In our observations, most of the real TFBSs had a positive correlations because with the positively correlated coding, the information that they contained decreased accordingly, but the information was transferred correctly.

Interestingly, we find that if there is a real TFBS encoded by one strand, then there often are peaks on both strands, but the peaks on the opposite strand are usually lower. We think this phenomenon happens for two reasons: first, certain factors bind to their TFBS by inserting a domain into the DNA grooves. In this case, both strands of DNA could have physical contact with the transcription factor; hence, both sides could transfer the regulatory information to the factor, which is detected by our method. Second, it is not known from which strand the background noise comes. Therefore, for example, for r = 2, the occurrence probability of AG equals TC. Therefore, the complementary strand of a real TFBS can have a high *TI*.

Furthermore, this information transmission model has the potential to be useful in other research areas, for example, in the computational identification of other motifs.

## Concluding remarks

In this work, we present a novel model for transcription factor binding regulatory DNA sites. This information transmission model is based on information theory and effectively incorporates position interdependencies. By testing the model on both real and artificial data sets, we have illustrated that our method is efficient at predicting unknown TFBSs.

## Materials and methods

### Data set preparation

The TFBSs of the 11 TFs and regulatory region sequences were obtained from the yeast *S. cerevisiae* Promoter Database (SCPD, http://rulai.cshl.edu/SCPD) [28]. This data set includes 68 regulatory regions with a total length of 30299 bp. These sequences harbour 309 experimentally mapped TFBS, including 141 real TFBSs of the 11 TFs. The chromosome sequences of *S. cerevisiae* were obtained from the National Center for Biotechnology Information (NCBI) reference sequence database.

The artificial sequences used in the test were randomly generated, taking into account the GC content of the *S. cerevisiae* genome. The pseudo-TFBSs of *gcn4*, *gal4* and *mcb* were randomly generated from PWMs. We did not generate the correlated TFBSs directly because it is difficult to make the pseudo-TFBSs conform to the correlation relationships, as real TFBSs do.

### Background probabilities calculation

Background probabilities are used to estimate the information carried by the TFBS before the binding event. An *L-*base window slides through the chromosomes, and all of the *r*-base sub-sequences in this window are counted. After the scanning, 4^r^ probabilities are calculated for all the 4^r^ possible *r*-base sub-sequences. This computation is time-consuming, but once the background probabilities are worked out, they can be reused in all of the TFBS predictions of this species without being recalculated.

### Sequence alignment

The TFBSs of the TFs were separately aligned by the ClustalW multiple alignment programme with the default argument, and the aligned TFBSs and the background probabilities were used to calculate the *MTI*.

### Computation environment

The novel method was implemented with a programme named tfbsInfoScaner, which was written in standard C++. This programme can be run on different computer platforms, and the full source code is available free for non-commercial use upon request by contacting the authors. Our test was run on a 64-CPU Altix 3700 server (Silicon Graphics, Mountain View, CA).

## Competing interests

The authors declare that they have no competing interests.

## Authors’ contributions

LL and JY formulated the study. MT, DY and YJ performed the research. LD analysed the data. YW and BL participated in analysis and discussion. MT drafted the manuscript. JY revised the manuscript. All authors read and approved the final manuscript.

## References

[B1] GuhaThakurta D Computational identification of transcriptional regulatory elements in DNA sequence Nucleic Acids Res 2006 34 3585 3598 16855295 10.1093/nar/gkl372 PMC1524905

[B2] Kono H Sarai A Structure-based prediction of DNA target sites by regulatory proteins Proteins 1999 35 114 131 10090291

[B3] Steffen NR Murphy SD Tolleri L Hatfield GW Lathrop RH DNA sequence and structure: direct and indirect recognition in protein-DNA binding Bioinformatics 2002 18 S22 S30 12169527 10.1093/bioinformatics/18.suppl_1.s22

[B4] Morozov AV Havranek JJ Baker D Siggia ED Protein-DNA binding specificity predictions with structural models Nucleic Acids Res 2005 33 5781 5798 16246914 10.1093/nar/gki875 PMC1270944

[B5] Siggers TW Honig B Structure-based prediction of C2H2 zinc-finger binding specificity: sensitivity to docking geometry Nucleic Acids Res 2007 35 1085 1097 17264128 10.1093/nar/gkl1155 PMC1851644

[B6] Berg OG von Hippel PH Selection of DNA binding sites by regulatory proteins. Statistical-mechanical theory and application to operators and promoters J Mol Biol 1987 193 723 750 3612791 10.1016/0022-2836(87)90354-8

[B7] Djordjevic M Sengupta AM Shraiman BI A biophysical approach to transcription factor binding site discovery Genome Res 2003 13 2381 2390 14597652 10.1101/gr.1271603 PMC403756

[B8] Mahony S Hendrix D Golden A Rokhsar DS Transcription factor binding site identification using the self-organizing map Bioinformatics 2005 21 1807 1814 15647296 10.1093/bioinformatics/bti256

[B9] Makita Y De Hoon MJ Ogasawara N Miyano S Nakai K Bayesian joint prediction of associated transcription factors in Bacillus subtilis Pac Symp Biocomput 2005 10 507 518 15759655 10.1142/9789812702456_0048

[B10] Kel AE Gossling E Reuter I Cheremushkin E Kel-Margoulis OV MATCH: A tool for searching transcription factor binding sites in DNA sequences Nucleic Acids Res 2003 31 3576 3579 12824369 10.1093/nar/gkg585 PMC169193

[B11] Cardon LR Stormo GD Expectation maximization algorithm for identifying protein-binding sites with variable lengths from unaligned DNA fragments J Mol Biol 1992 223 159 170 1731067 10.1016/0022-2836(92)90723-w

[B12] Lawrence CE Altschul SF Boguski MS Liu JS Neuwald AF Detecting subtle sequence signals: a Gibbs sampling strategy for multiple alignment Science 1993 262 208 214 8211139 10.1126/science.8211139

[B13] Hughes JD Estep PW Tavazoie S Church GM Computational identification of cis-regulatory elements associated with groups of functionally related genes in Saccharomyces cerevisiae J Mol Biol 2000 296 1205 1214 10698627 10.1006/jmbi.2000.3519

[B14] Schneider TD Stormo GD Gold L Ehrenfeucht A Information content of binding sites on nucleotide sequences J Mol Biol 1986 188 415 431 3525846 10.1016/0022-2836(86)90165-8

[B15] Stormo GD Fields DS Specificity, free energy andinformation content in protein-DNA interactions Trends Biochem Sci 1998 23 109 113 9581503 10.1016/s0968-0004(98)01187-6

[B16] Benos PV Probabilistic code for DNA recognition by proteins of the EGR family J Mol Biol 2002 323 701 727 12419259 10.1016/s0022-2836(02)00917-8

[B17] Bulyk ML Johnson PL Church GM Nucleotides of transcription factor binding sites exert interdependent effects on the binding affinities of transcription factors Nucleic Acids Res 2002 3 1255 1261 11861919 10.1093/nar/30.5.1255 PMC101241

[B18] Man T-K Stormo GD Non-independence of Mnt repressor–operator interaction determined by a new quantitative multiple fluorescence relative affinity (QuMFRA) assay Nucleic Acids Res 2001 29 2471 2478 11410653 10.1093/nar/29.12.2471 PMC55749

[B19] Udalova IA Quantitative prediction of NF-kappa B DNA-protein interactions Proc Natl Acad Sci USA 2002 99 8167 8172 12048232 10.1073/pnas.102674699 PMC123039

[B20] Wolfe SA Analysis of zinc fingers optimized via phage display: evaluating the utility of a recognition code J Mol Biol 1999 285 1917 1934 9925775 10.1006/jmbi.1998.2421

[B21] Barash Y Modeling dependencies in protein-DNA binding sites Proceedings of RECOMB-03 2003, 28 37

[B22] Zhao X Finding short DNA motifs using permuted Markov models J Comput Biol 2005 12 894 906 16108724 10.1089/cmb.2005.12.894

[B23] Ellrott K Identifying transcription factor binding sites through Markov chain optimization Bioinformatics 2002 18 Suppl. 2 S100 S109 12385991 10.1093/bioinformatics/18.suppl_2.s100

[B24] Marinescu VD MAPPER: a search engine for the computational identification of putative transcription factor binding sites in multiple genomes BMC Bioinforma 2005 6 79 10.1186/1471-2105-6-79 PMC1131891 15799782

[B25] King OD Roth FP A non-parametric model for transcription factor binding sites Nucleic Acids Res 2003 31 e116 14500844 10.1093/nar/gng117 PMC206482

[B26] Zhou Q Liu JS Modeling within-motif dependence for transcription factor binding site predictions Bioinformatics 2004 20 909 916 14751969 10.1093/bioinformatics/bth006

[B27] Tomovic A Oakeley EJ Position dependencies in transcription factor binding sites Bioinformatics 2007 23 933 941 17308339 10.1093/bioinformatics/btm055

[B28] Bussemaker HJ Li H Siggia ED Regulatory elementdetection using correlation with expression Nature Genet 2001 27 167 171 11175784 10.1038/84792

[B29] Cooper GM Sidow A Genomic regulatory regions:insights from comparative sequence analysis Curr Opin Genet Dev 2003 13 604 610 14638322 10.1016/j.gde.2003.10.001

[B30] Defrance M Touzet H Predicting transcription factor binding sites using local over-representation and comparative genomics BMC Bioinforma 2006 7 396 10.1186/1471-2105-7-396 PMC1570149 16945132

[B31] Blanchette M Bataille AR Chen X Poitras C Laganiere J Genome-wide computational prediction of transcriptional regulatory modules reveals new insights into human gene expression Genome Res 2006 16 656 668 16606704 10.1101/gr.4866006 PMC1457048

[B32] Aerts S Van Loo P Thijs G Moreau Y De Moor B Computational detection of cis-regulatory modules Bioinformatics 2003 19 II5 II14 14534164 10.1093/bioinformatics/btg1052

[B33] Jegga AG Gupta A Gowrisankar S Deshmukh MA Connolly S CisMols analyzer: identification of compositionally similar cis-element clusters in ortholog conserved regions of coordinately expressed genes Nucleic Acids Res 2005 33 W408 W411 15980500 10.1093/nar/gki486 PMC1160246

[B34] Shannon CE A mathematical theory of communication (Part 1) Bell System Technical Journal 1948 27 379 423

[B35] Shannon CE A mathematical theory of communication (Part 2) Bell System Technical Journal 1948 27 623 656

[B36] Zhu J Zhang MQ SCPD: a promoter database of the yeast Saccharomyces cerevisiae Bioinformatics 1999 15 607 611 10487868 10.1093/bioinformatics/15.7.607

[B37] Bailey TL Elkan C Fitting a mixture model by expectation maximization to discover motifs in biopolymers Proc Int Conf Intell Syst Mol Biol 1994 2 28 36 7584402

